# Phenotypic convergence of artificially reared and wild trout is mediated by shape plasticity

**DOI:** 10.1002/ece3.3156

**Published:** 2017-06-22

**Authors:** Jorge Ruben Sánchez‐González, Alfredo G. Nicieza

**Affiliations:** ^1^ Department of Biology of Organisms and Systems University of Oviedo Oviedo Spain; ^2^ UMIB Research Unit of Biodiversity (UO‐CSIC‐PA) Mieres Spain

**Keywords:** ecological convergence, environmental conditioning, field experiment, habitat complexity, morphological variability, phenotypic plasticity, *Salmo trutta*, stream

## Abstract

Phenotypic plasticity can be viewed as the first level of defense of organism homeostasis against environmental stress and therefore represents the potential to deal with rapid environmental changes. Transitions between low complexity, artificial environments and complex, natural habitats can promote phenotypic plasticity. Here, we conducted an experimental introduction with juvenile brown trout to evaluate the plasticity of shape in response to a transition between contrasting environments. We released 202 juvenile trout reared under hatchery conditions in a natural stream and analyzed changes in shape and morphological variability after 5 months. A geometric morphometrics approach based on 14 landmarks was used to compare changes in body shape for 37 fish recaptured at the end of the experiment. A similar number of hatchery and wild fish caught at the receptor stream were used as controls for shape in the two environments. After 5‐months, fish showed significant change in shape, shifting from elongated to robust shapes, and affecting to the relative position of the caudal peduncle. These new shapes were closer to wild than to the hatchery shapes, suggesting a process of rapid phenotype change. Moreover, these changes were concomitant with a marked increase in morphological variability. Our results support the hypothesis that phenotypic plasticity is a major potential for adjustment to environmental change but not the idea that shape can be constrained by initial shapes. We confirmed the “increased” variance hypothesis and phenotype convergence with wild morphs. This has important implications because stresses the role of phenotypic plasticity as a buffer that allows organisms to cope with important environmental discontinuities at time scales that preclude the onset of adaptive adjustments. We suggest that environmental conditioning and shape plasticity can overcome both reduced morphological diversity and phenotype uncoupling with habitat characteristics resulting from initial rearing in low complexity artificial environments.

## 1 INTRODUCTION

A central question in forecasting the viability of organisms and populations to rapid environmental change is whether phenotypic plasticity can mediate the adjustment to the new conditions before an evolutionary response is attained. The ability to plastically altering phenotypes is an effective way to deal with rapid change, facilitating organism persistence, and further evolution of adaptive responses (Thompson, 1991). Phenotypic plasticity can also act as a buffer for environmental variation that, otherwise, might promote local adaptation. Thus, phenotypic plasticity itself can be an adaptive trait exposed to natural selection (Price, Qvarnström, & Irwin, 2003; Thompson, 1991).

Local populations and ecotypes often differ in body shape because of genetics (Hard, Winans, & Richardson, 1999; Nicieza, 1995; Taylor, 1991), environmental conditioning (Langerhans, Layman, Langerhans, & Dewitt, 2003; Svanbäck & Eklöv, 2002), or both (Keeley, Parkinson, & Taylor, 2007; Marcil, Swain, & Hutchings, 2006). In aquatic environments, body shape of active swimmers can reflect morphological adjustments to habitat characteristics. In general, elongated bodies confer a higher performance for endurance and sustained swimming, whereas deeper bodies can promote higher performance in burst swimming (Langerhans & Reznick, 2010; Morinville & Rasmussen, 2008; Ojanguren & Braña, 2003). Moreover, the observed morphological differentiation between wild and domesticated strains suggests that correspondences between body shapes and habitats or locomotion styles could be the result of adaptive processes (Jonsson & Jonsson, 2011; Von Cramon‐Taubadel, Ling, Cotter, & Wilkins, 2005). However, besides genetic variation, the environmental differences between natural and artificial environments are expected to exert a determinant influence on fish shape (Bohlin, Sundstrom, Johnsson, Hojesjo, & Pettersson, 2002; Jonsson & Jonsson, 2011; Vehanen & Huusko, 2011).

Most economically important freshwater and anadromous fish are subject to restocking and enhancement of their populations. However, the morphological changes experienced by fish reared in artificial environments can have negative effects for survival in the wild (Bohlin et al., 2002; Von Cramon‐Taubadel et al., 2005). Thus, the effectiveness of stocking with artificially reared fish might depend on their ability to converge with the “environmental” phenotype. Such convergence, mediated by phenotypic plasticity, might compensate for differences associated with genetic or environmental variation, and therefore, it can have profound implications for population reinforcement.

Habitat complexity can be an important source of morphological diversity. Streams are spatially complex environments, with a high diversity of microhabitats with regard to size, depth, current speed, slope, or turbulence regimes (Ward, Tockner, Arscott, & Claret, 2002). In addition, flow regimes, dominant food sources (*e.g*., benthos *vs*. drift), and fish densities can change drastically over time. In contrast, extremely low spatial complexity and uniformity of flow, diet, and fish densities are characteristics of hatchery environments that can affect swimming routines. Because fish shape can be molded by environmental conditions affecting exercise demands and swimming styles, artificial habitats are expected produce a reduced range of shapes compared to natural streams. Here, we assume individual variation in fish behavioral decisions; this individual variation can be expressed in spatially heterogeneous environments, but not in low complexity hatchery environments where the range of hydrodynamic niches is strongly reduced.

**Figure 1:**
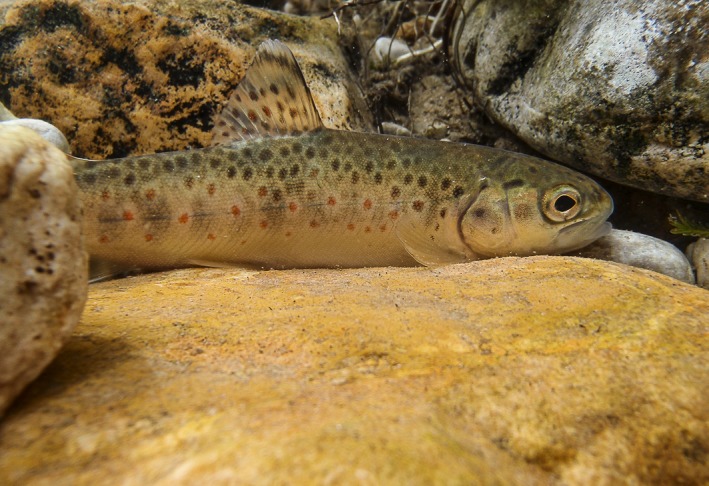
Juvenile brown trout, *Salmo trutta*, in a zero‐flow site on the stream bed

We conducted a field experiment with brown trout (*Salmo trutta* Linnaeus 1758; Figure 1) to explore whether juvenile shape is continuously molded by the environmental conditions via phenotypic plasticity or, in contrast, it is constrained by the shape configurations attained at earlier stages. First, we test the hypothesis that hatchery‐reared juveniles will undergo remarkable changes in body shape after the transition to a natural stream. If so, we test two nonexclusive hypotheses: (1) the change in shape will be consistent with the shape configurations of wild fish (convergence hypothesis), and (2) the phenotypic change results from the spread of initial shape configurations in response to dispersion of fish across a greater variety of microhabitats (increased variance hypothesis).

## 2 MATERIALS AND METHODS

### 2.1 Experimental fish

The experimental fish were hatchery‐reared juvenile trout (age 0+) obtained from wild parents caught in the river Sella (Asturias, northern Spain). A total of 30 males and 30 females were used to produce several thousands of embryos that were reared in the river Espinareu hatchery (Centro Ictiogénico de Infiesto, Principality of Asturias). Three months after hatching, we transferred 250 juvenile fish to the University of Oviedo facilities, where they were kept in six circular, 80‐L PE tanks (about 40 fish per tank), with a constant input flow of 12 L/min, and fed *ad libitum*. Temperature was set at 14.0 ± 2.0°C.

By the end of August, a total 202 hatchery‐reared fish were photographed, individually marked with unique VIE codes (Visible Implant Elastomer, NorthWest Marine Tech., WA, USA), weighed to the nearest 0.01 g, and then released in river Santianes (Appendix S2). At the time of stocking (1st September), the fish were 8‐months old. River Santianes is a low elevation (30–280 m.a.s.l.), small headwater stream (Strahler order 1), tributary of the river Sella. The stream structure is highly heterogeneous and consists of small pools, waterfalls, riffles, and runs (Appendix S2). Thus, it combines sections with fast flow and others with very low water speed. Channel sinuosity is relatively high, and water flows are usually under 0.1 m^3^/s in summer. Canopy cover is near 100% in most of the channel, so there is abundant debris that contributes to higher spatial heterogeneity and diversity of microhabitats. The European eel (*Anguilla anguilla*) is the only fish species that co‐occurs with trout in the river Santianes.

After 5 months, we conducted an electrofishing operation (315 V, 60 Hz, 3 ms) with a BSE model EFGI‐650 electrofisher (Bretschneider‐Spezialelektronik, Chemnitz, Germany) along a reach of 1,200 m (500–600 m downstream and upstream of the point of release) to capture the hatchery‐reared fish and a sample of wild fish. A total of 37 hatchery‐reared and 42 wild trout were captured. All the fish were anesthetized with Benzocaine (Ethyl 4‐aminobenzoate; Sigma Aldrich, Darmstadt, Germany; product number: E1501, final concentration: 50 mg/L), identified by reading of their unique VIE codes, weighed, and photographed. To ensure the consistency of morphological landmarks, fish were carefully aligned on their right side prior to take the pictures. Then, their left side was photographed with a Sony Cyber‐shot DSC‐H2 digital camera. Images were taken using a standardized method according to which all fish were placed on a grid board in a relaxed position and checked carefully for linearity of the midline in order to avoid any arching effect (Valentin, Penin, Chanut, Sevigny, & Rohlf, 2008); all cases presenting some indication of curvature were discarded.

To test the hypothesis that morphological convergence can be mediated by phenotypic plasticity, we conducted multiple comparisons of body shapes between hatchery‐reared fish at the start of the experiment (before release, hereafter HAT_before_), hatchery fish recaptured in the river Santianes 5‐months after the release (HAT_after_), and wild fish from the river Santianes (WILD). The hatchery fish used here and the Santianes wild trout belong to the same genetic unit (Nicieza, Alfredo G., Choda, Magda, García, Susana, Cano, Jose Manuel, Sánchez‐González, Jorge R., & Álvarez, David). HAT_after_ fish were part of the 202 fish released at Santianes river at the start of the experiment. To ensure data independence, we haphazardly selected 40 individuals from the pool of HAT_before_ fish, after excluding the 37 HAT_after_ fish (those 37 fish recaptured in February). As a result, we used a total of 119 individual fish (HAT_after_: *n* = 37; WILD: *n* = 42; HAT_before_: *n* = 40).

In addition, we conducted a longitudinal analysis to test the hypothesis that body shape is highly reactive to the physical conditions fish encounter in their environment, by comparing the shape of the same 37 individuals before release in Santianes river (HAT_before_) and at the time of recapture 5‐months later (HAT_after_).

### 2.2 Morphometrics

**Figure 2:**
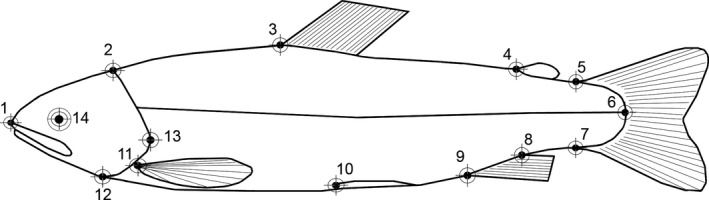
Landmarks used for morphometric analyses: (1) tip of upper jaw; (2) posterior supraoccipital notch, (3) anterior insertion of dorsal fin; (4) origin of adipose fin; (5 and (7) anterior junction of dorsal and ventral membrane from caudal fin; (6) intersection of lateral line and membrane of caudal fin; (8 and (9) posterior and anterior insertion of anal fin; (10) origin of pectoral fin; (11) origin of pelvic fin; (12) ventral insertion between operculum and the body outline; (13) posterior tip of operculum; (14) centre of orbital (modified from Von Cramon‐Taubadel et al., 2005; Keeley et al., 2007)

We selected a total of 14 landmarks (Figure 2); of these, 12 landmarks had an unequivocal anatomic significance, and two landmarks (7 and 13) were geometrically determined but presented a clear anatomical undertone. Landmarks were obtained by using tpsDig 2.17 (Rohlf, 2013a). Landmark configurations were superimposed, aligned, scaled, and rotated to a *consensus* shape using two thin‐plate Spline analyses (Zelditch, Swiderski, Sheets, & Fink, 2004). The first analysis was conducted on 119 fish belonging to the three experimental groups. The second, on the 74 shapes derived from the 37 hatchery fish recaptured at the end of the experiment. These analyses were carried out with tpsRelw version 1.53 (Rohlf, 2013b) to obtain 24 partial warp scores (22 uniform and 2 non‐uniform components of shape). Centroid sizes were obtained using tpsRelw version 1.53 and used as a proxy of body size.

### 2.3 Statistical analyses

As part of the longitudinal analysis, we conducted a principal component analysis on the covariance matrix to extract the major components of shape variation from the original shape variables (partial warp scores; alpha = 0). We used analysis of variance with repeated measures to analyze the effects of exposure to stream conditions on fish shape (the three‐first components of the PCA were used as dependent variables on separate ANOVAs). Time was the within‐subjects factor (initial and final shapes) and tests were based on the multivariate approach. To infer allometric constraints in shape plasticity, we examined (1) the relationships between final and initial shape for each component, and (2) the relationships between final and initial size. In addition, we examined the relationship between somatic growth (change in centroid size) and shape plasticity (measured either as the absolute change in PC1, or the Euclidean distance “before‐after” using the first five principal components.

In a second step, we compared shapes of the three experimental groups (HAT_before_, HAT_after_, and WILD) to test the hypothesis of convergence in shape between HAT_after_ and WILD individuals. Again a covariance‐based PCA was carried out to generate an empirical morphospace (alpha = 0) with orthogonal shape variables. To check for morphological differences among HAT_after_, HAT_before_, and WILD fish, we conducted ANOVAs on the two first components of the PCA. Finally, we performed a discriminant analysis (DA) and calculated squared Mahalanobis distances between the different groups. To discard potential problems associated with small sample sizes, cross‐validation of the discriminant functions was performed by random resampling of the experimental individuals; a subsample containing 90% of the cases was used for analysis, and the remainder 10% were the holdout sample. This second DA was carried out to construct discriminant functions based on a larger number of cases in a loop of 1,000 random samples. We used Levene's test to check for homogeneity of variances and Box's M statistic to test for the homogeneity of the dispersion matrices. Multivariate normality was tested using Shapiro–Wilk's multivariate test from the *R package mvnormtest* (Jarek, 2012). Morphometric data met the assumptions of homogeneity of variances (*p *>* *.05) and normality.

Finally, we conducted an ANCOVA of PC1 with centroid size (log‐transformed) as a covariate to analyze the relationship between shape and size. ANCOVA was performed after checking for the homogeneity of regression slopes and the homogeneity of variances (Levene test). Differences in fish size (centroid size) were evaluated using ANOVA tests or the nonparametric alternatives (Kruskal–Wallis H and Welch *t*‐test for unequal variances). We used Tukey's *post hoc* tests to identify homogeneous groups after significant ANOVA. All statistical analyses were carried out using the R version 3.1.2 (R Development Core Team 2014) except otherwise specified.

## 3 RESULTS

### 3.1 Temporal variation of shape

**Figure 3:**
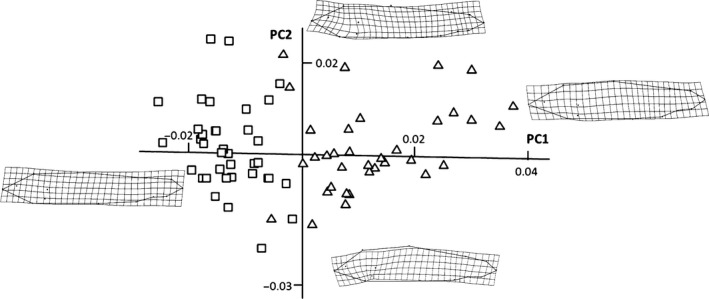
Deformation grids (obtained from tpsSpline version 1.20 by Rohlf, 2004) and PC scores for hatchery‐reared fish at the time of release (HAT_before_; □) and after a 5‐months period of residency in Santianes river (HAT_after_; △)

After 5 months in the Santianes river, HAT_after_ fish showed a significant increase in size (CS: paired *t*‐test, *t*_36_ = −10.49, *p *<* *.000001). The first two components of the PCA extracted 45.8% of total variation in shape. Scores for the recaptured fish before and after release were clearly segregated across PC1 (31.4% of total variance; Figure 3). Before release (August; HAT_before_) all the fish had negative scores for PC1, whereas all but three of the February scores (HAT_after_) were ordinated on the right side of PC1. HAT_after_ fish had more robust shapes resulting from a shortening of their caudal peduncle and a backward displacement of the anterior landmarks (Figure 3).

**Figure 4:**
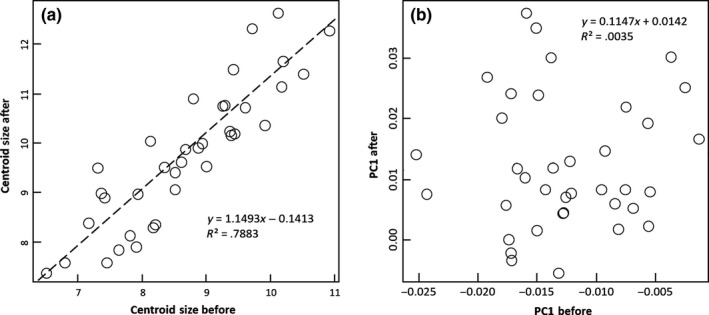
Regressions of (a) centroid sizes and (b) PC1 scores of hatchery‐reared fish before and after the release in Santianes river. The data correspond to hatchery fish recaptured at the end of the experiment (*N* = 37), after a period of 5 months in the river

We found a significant effect of time on PC1 (Wilk's Lambda = 0.177, *F*_1,36_ = 167.55, *p *<* *.000001) but not for PC2 (Wilk's Lambda = 0.987, *F*_1,36_ = 0.4955, *p *=* *.488) or PC3 (Wilk's Lambda = 0.932, *F*_1,36_ = 2.64, *p *=* *.113). As expected, initial size explained a large part of variation in final size ($R_{\mathrm{adj}}^{2}$ = 0.78, *F*_1,35_ = 130.30, *p *<* *.000001; Figure 4a). In contrast, final shapes were statistically unrelated to initial shapes (PC1; $R_{\mathrm{adj}}^{2}$ = −0.02, *F*_1, 35_ = 0.12, *p *=* *.728; Figure 4b). We assayed the regressions between final and initial states for the first 10 components of the PCA, but none of these were significant after adjustment of significance levels with the Bonferroni sequential criterion (only for PC5 we obtained a significant value (*p *=* *.02) prior to Bonferroni's correction), which suggests the absence of strong constraints on shape plasticity. In addition, Euclidean distances between initial and final shape configurations were not related to growth rates: growth explained a nonsignificant fraction of the temporal variation in PC1 ($R_{\mathrm{adj}}^{2}$ = −0.042, *F*_1, 35_ = 0.85, *p *=* *.363) or Euclidean distances (PC1 and PC2: $R_{\mathrm{adj}}^{2}$ = 0.032, *F*_1, 35_ = 2.20, *p *=* *.14; PC1 to PC5: $R_{\mathrm{adj}}^{2}$ = 0.051, *F*_1, 35_ = 2.91, *p *=* *.096).

### 3.2 Wild versus artificially reared fish

**Figure 5:**
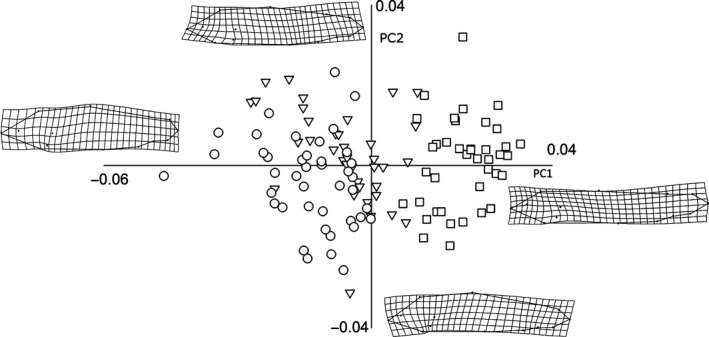
Deformation grids (tpsSpline version 1.20 by Rohlf, 2004) and scores for PC1 and PC2 showing shape differences and similarities for hatchery‐reared fish at the start of the experiment (HAT_before_, □), hatchery fish after 5‐months in the river (HAT_after_, ▽), and wild fish (WILD, ○)

HAT_after_ fish were smaller than WILD but bigger than HAT_before_ fish (Welch's test, *F*_2,61_ = 50.15, *p *<* *.000001; Appendix S1). The first three principal components explained 57.47% of the total variance in shape. PC1 (33.59%) ordered the specimens according to fish category and body depth. Wild fish scored negative values of PC1 (bigger body depth and a lower head position), whereas hatchery fish had positive values on the right side of PC1 (elongated shapes: Figure 5). Like WILD trout, most of the HAT_after_ fish had negative scores. For PC1, ANOVA and Tukey's post hoc tests indicated significant differences among WILD, HAT_before_ and HAT_after_ fish (*F*_2,116_ = 162.96; *p *<* *.000001). Along PC2 (14.54%) individuals were ordered according to the relative position of the tail. Fish with positive scores had a higher relative position of the tail, whereas low tail individuals scored negative values (Figure 5). ANOVA indicated differences among groups (*F*_2,116_ = 3.81; *p *=* *.025); PC2 scores were lower for WILD than for HAT_after_ fish (Tukey post hoc test; *p *<* *.05). HAT_before_ fish did not differ from HAT_after_ (*p *=* *1.000) or WILD fish (*p *=* *.28).

**Figure 6:**
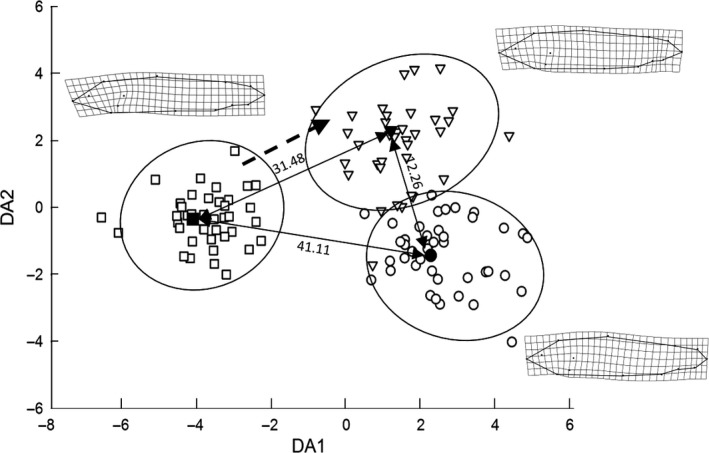
Results of the discriminant analysis conducted on morphometric variables for hatchery‐reared fish at the start of the experiment (HAT_before_, □; *n* = 40), a different group of hatchery fish after 5‐months in the river (HAT_after_, ▽; *n* = 37), and wild fish (WILD, ○; *n* = 43). Double‐headed arrows and values indicate Squared Mahalanobis distances between group centroids. The dashed arrow indicates the morphological trajectory from initial to final morphologies after a 5‐months period of residency in the river. Landmark configurations and deformation grids (tpsSpline version 1.20 by Rohlf, 2004) of consensus fish were obtained from group centroids

We conducted a DA by calculating prior probabilities for HAT_before_, HAT_after_, and WILD groups (0.3361, 0.3109, and 0.3529, respectively). DA functions successfully classified 93.33% of the fish, with 100% accuracy for HAT_before_, 86.36% accuracy for HAT_after_ and with 92.86% accuracy for the WILD trout (Wilks’ Lambda: 0.039, *F*_48,186_ = 15.74, *p *<* *.00001). Squared Mahalanobis Distances (SMD) revealed that HAT_before_ fish were clearly differentiated from WILD (SMD: 41.1) or HAT_after_ fish (SMD: 31.5), whereas WILD and HAT_after_ morphospaces were much closer (SMD: 12.2) (Figure 6). In the second DA (a loop of 1,000 random samples), we obtained an overall accuracy of 90.7%. Again, classification success was higher for HAT_before_ fish (98.3%) than for HAT_after_ and WILD fish (86.0% and 88.5%, respectively). These results suggest that morphological diversity was higher for HAT_after_ and WILD fish than for HAT_before_ fish. In fact, mean Euclidean distances for the three groups supported the increased variance hypothesis (HAT_before_: 0.022; HAT_after_: 0.025, WILD: 0.026).

**Figure 7:**
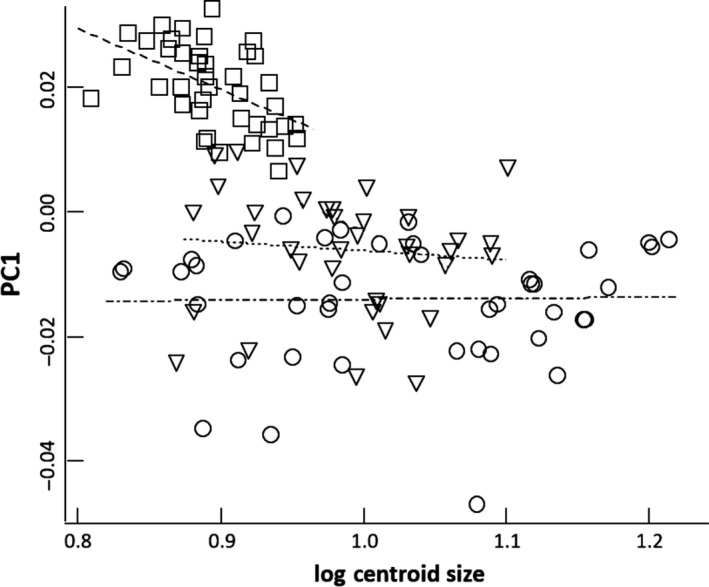
Relationships between shape (PC1) and fish size (centroid size) in hatchery‐reared (HAT_before_, □, dashed line), hatchery fish after 5‐months in the river (HAT_after_, ▽, dotted line), and wild fish (WILD, ○, dotted and dashed line)

An ANCOVA of shape (PC1) with size (CS) as a covariate revealed significant differences in regression slopes (ANCOVA; *F*_2,113_ = 3.13, *p *=* *.048). Interestingly, there was not a significant difference between the slopes for WILD and HAT_after_ fish (*F*_1,75_ = 0.35, *p *=* *.555), and moreover, these slopes were close to zero (WILD: *F*_1,40_ = 0.01, *p *=* *.92; HAT_after_: *F*_1,375_ = 0.32, *P *=* *.57; Figure 7). However, the differences in shape between HAT_after_ and WILD fish remained significant after adjusting for body size (ANCOVA; *F*_1,76_ = 12.64, *p *=* *.0007). In contrast, HAT_before_ fish showed a clear negative, significant slope (*F*_1,38_ = 13.05, *p *=* *.0009; Figure 7) that was different from those of WILD (*F*_1,78_ = 6.90, *p *=* *.010) and HAT_after_ (*F*_1,73_ = 4.68, *P *=* *.034) fish.

## 4 DISCUSSION

The introduced trout exhibited a rapid change of body shape, parallel to an increment in morphological variability. These results supported both the convergence and the increased variance hypotheses: (1) growth under “natural stream” conditions can promote shape convergence between hatchery‐reared fish and wild fish, and (2) fish living in spatially complex environments will exhibit higher shape variability than fish living in low complexity, artificial environments.

Dorsoventral dimensions and head shape accounted for most of the variation in shape, and the observed changes conferred the released fish a more robust form (“bull” shape: deeper bodies, shorter heads and anterior regions, and shorter and thicker peduncles) than hatchery trout. In agreement with the hypothesis of convergence, these changes were directional, delineating a trajectory from hatchery to wild phenotypes. River conditions (*i.e*., hydrological conditions, relative abundance of benthos *vs*. drift prey, stream bed structure, or fish densities) can conform a heterogeneous mosaic of microhabitats that, moreover, change drastically throughout the year. Therefore, the observed increase in morphological diversity was consistent with the exposure to a greater environmental complexity derived from this dynamic mosaic. This is also consistent with the idea that the observed changes resulted from phenotypic plasticity in response to the dispersion of fish across a great variety of microhabitats.

Regression and covariance analysis of slopes did allow us to discard the hypothesis that changes in shape experienced by trout after release in a natural stream might have been the result of allometric processes only. First, the lack of correlation between shape change and growth does not support a major role of allometric processes. Second, we found no evidence that final shape can be constrained by initial shape, which again is not consistent with an allometric effect. Third, although HAT_before_ fish showed a significant negative relationship between shape (PC1) and size, size and shape were clearly uncorrelated for both WILD and HAT_after_ fish. Furthermore, we compared the shape of same‐size fish from these three groups (log‐CS greater than 0.85 and lower than 1.00; see Figure 7). Clearly, HAT_before_ fish scored much higher values of PC1 than WILD or HAT_after_ fish, and the difference keeps unambiguous when the comparison is restricted to the size range 0.90 – 0.95 (Figure 6). Thus, the observed changes in shape cannot be explained by size variation, which supports the hypothesis that fish shape is highly plastic and can be molded by environmental conditions. Habitat characteristics and flow conditions can shape fish morphology through its effects on locomotion styles and exercise intensity. Hence, the observed changes could be mostly the outcome of the new conditions faced by fish in the stream (Svanbäck & Eklöv, 2002, 2004).

Hatchery‐reared salmonids are exposed to rather uniform and sustained currents where fish are forced to perform sustained swimming, and this could favor the development of more elongated and “endurance‐adjusted” morphologies (Langerhans & Reznick, 2010; Ohlberger, Staaks, & Hölker, 2006; Rouleau, Glémet, & Magnan, 2009; Vehanen & Huusko, 2011). In contrast, stream fishes are expected to face a wider range of situations, combining burst swimming and endurance, and this can promote the development more robust shapes. In our study, trout were also ordered according to the shape of their caudal peduncle; wild fish had shorter and thicker peduncles, which again is associated with burst swimming performance (Langerhans & Reznick, 2010; Morinville & Rasmussen, 2008). In this line, a number of studies have reported substantial differences in morphology between wild and captive‐reared salmonids, although the results were not always consistent. For example, captive adult coho salmon had deeper trunks compared to wild salmon (Hard et al., 2000). In Atlantic salmon, hatchery juveniles released in a river rapidly diverged in shape from the hatchery phenotype (Stringwell et al. 2014). As we have reported here for juvenile trout, these changes involved increases in head size and caudal peduncle thickness. However, compared with their hatchery controls, survivors become more streamlined (Stringwell et al. 2014). Apparently, this contrasts with our finding that juvenile trout showed an increase in body depth after release in the river. This difference can be related to behavioral differences between the two species (drift *vs*. benthos feeding, use of fast *vs*. slow waters, etc.), or due to habitat differences in the experimental streams, that can affect the times of sustained versus burst swimming. However, the most interesting point is that the results for Atlantic salmon and brown trout were consistent in the direction of the morphological shift. In both cases, the changes in morphology showed by the released fish resulted in a phenotypic convergence toward the wild phenotypes (Stringwell et al. 2014 present study). That is, the shape trajectories of the released fish had the same ecological direction in the two study systems.

The results for PC2 reinforced our previous interpretation, as trout were ordered according to the relative position of their caudal peduncle, and lower caudal peduncles have been associated with the use of benthonic microhabitats (see Andersson, Johansson, Sundbom, Ryman, & Laikre, 2016). In contrast with the Atlantic salmon, which is predominantly a drift feeder, juvenile, and adult brown trout often feed on the benthos fauna (Andersson et al., 2016; Jonsson & Jonsson, 2011; Klemetsen et al., 2003). Compared to wild fish, hatchery‐reared fish showed more elongated bodies, and thinner and higher peduncles. These characteristics conform well to the conditions for swimming in artificial environments. In contrast, fish will acquire robust body shapes in environments where burst swimming outweighs stamina swimming as the main locomotion mode (Langerhans & Reznick, 2010), such as structurally complex habitats, low‐flow environments, and areas with a high level of predation.

Our longitudinal study (initial *vs*. final shape of recaptured fish) indicated that morphological changes leading to convergence with native fish were mediated by phenotypic plasticity, and not as result of selection for wild‐like phenotypes. However, as we have no information on shape of those fish that die or migrate, we cannot discard that fish recaptured at the end of the experiment (survivors and stayers) had a greater capability for plastic morphological change (*i.e*., the occurrence of non‐random selection on shape plasticity). Both of these outcomes are equally interesting because can have important consequences for management practices and the response to directional alterations of stream hydrology associated with climate change. In this context, an important question to be explored in the future research is whether the retention of a high degree of phenotypic plasticity for shape does differ among wild populations. More important, our results pointed out to the need for the exploration of shape plasticity in hatchery stocks (*i.e*., maintained under artificial rearing for several generations) because there is a risk that shape plasticity has been lost due to artificial selection or “natural” selection under hatchery conditions. In fact, a number of studies have reported that captively reared adults are competitively inferior and have lower reproductive success than wild adults (Berejikian et al., 2001; Fleming & Einum, 1997; Fleming, Lamber, & Jonsson, 1997; Jonsson & Jonsson, 2006; Neff, Garner, Fleming, & Gross, 2015). Therefore, the results obtained here for hatchery‐reared trout from a wild origin might be not directly applicable to multigeneration hatchery stocks.

## AUTHOR CONTRIBUTIONS

JRSG and AGN planned the work and conducted the sampling. JRSG carried out fish husbandry, processed the images, generated the morphometric, and analyzed the data. AGN contributed to statistical analyses. JRSG and AGN led the writing of the manuscript. All authors contributed critically to the drafts and gave final approval for publication.

## DATA ACCESSIBILITY

Data deposited in Figshare at https://doi.org/10.6084/m9.figshare.4968128.
