# Supplementary material for: Phenotypic convergence of artificially reared and wild trout is mediated by shape plasticity
Source: Ecol Evol. 2017 Jun 22;7(15):5922–9. doi: 10.1002/ece3.3156 (PMC5551095; doi:10.1002/ece3.3156)
Supplement: Supplementary file 1 [file ECE3-7-5922-s001.docx]

**Appendix S1.**

**Table S1.** Estimates for several indicators of size (mass, body depth, fork-length, centroid size; mean ± 1SD) for hatchery fish at the start of the experiment (HAT_before_), a different sample of hatchery fish after 5-mo in the river (HAT_after_), and wild fish at the recapture time (WILD); letters in bold indicate homogeneous groups at *P*<0.05 (Tukey *post hoc* test).

|  | ***n*** | **Mass (mg)** | **Body depth (mm)** | **Fork length (mm)** | **Centroid size** |
| --- | --- | --- | --- | --- | --- |
| HAT_before_ | 40 | 27.4±7.19 **C** | 11.55±1.23 **C** | 67.6±5.39 **C** | 7.87 ±0.62 **C** |
| HAT_after_ | 37 | 68.4±28.92 **B** | 16.12±2.70 **B** | 85.1±11.59 **B** | 9.81±1.41 **B** |
| WILD | 42 | 109.9±71.86 **A** | 18.89±4.90 **A** | 95.0±22.66 **A** | 10.97±2.73 **A** |

**Appendix S2.**

**Photography S2.** Typical section of the river Santianes, close to the point of release of the experimental fish.
