# Supplementary figures and images for: Phenotypic convergence of artificially reared and wild trout is mediated by shape plasticity
Source: Ecol Evol. 2017 Jun 22;7(15):5922–9. doi: 10.1002/ece3.3156 (PMC5551095; doi:10.1002/ece3.3156)

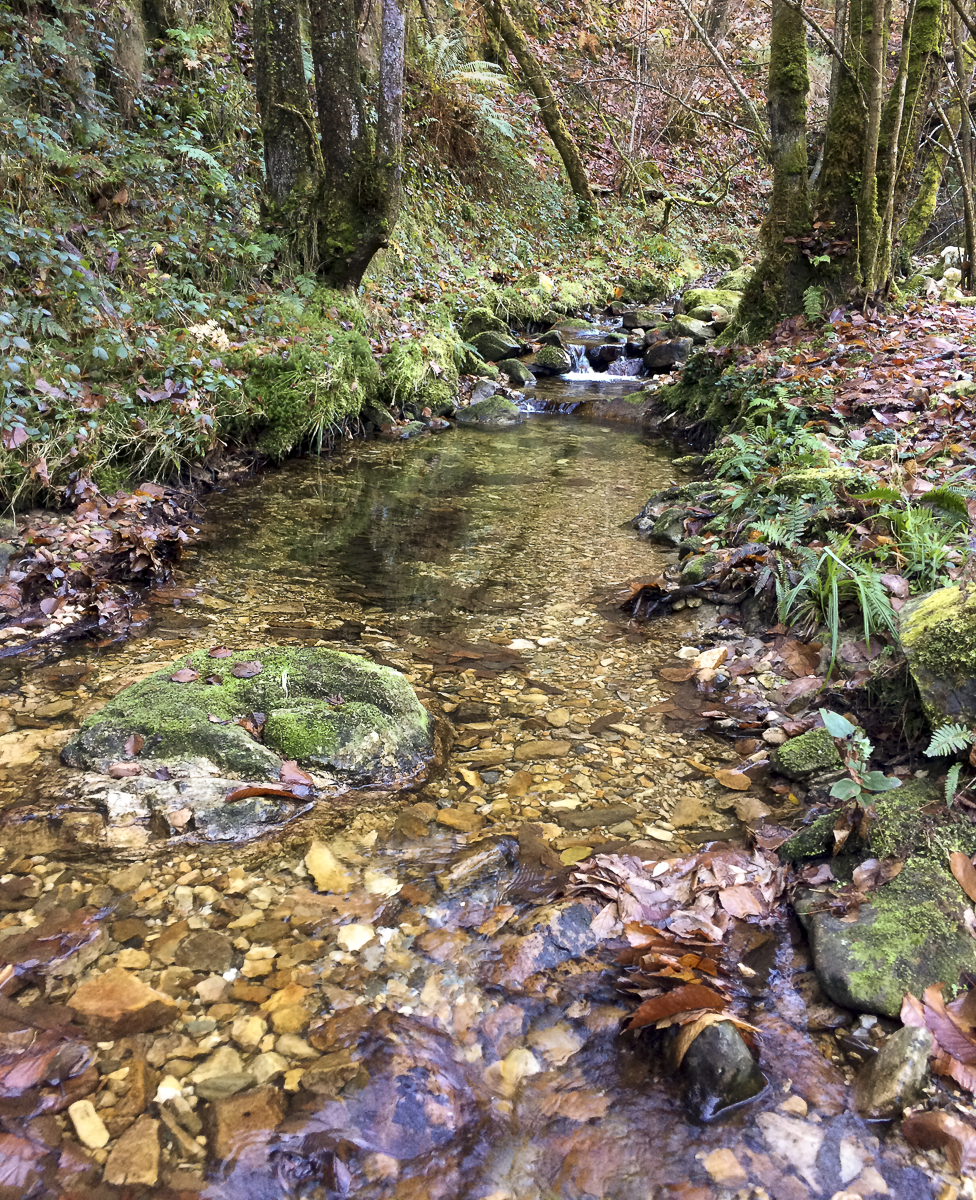

Supplement: Supplementary file 2 [file ECE3-7-5922-s002.tiff]
